# Enhancing Reflective Practice Using Prompts in Online Submission of Case Reports (OSCAR): An Exploratory Study Among Medical Students in Rural Longitudinal Integrated Clerkships

**DOI:** 10.5334/pme.1416

**Published:** 2024-12-26

**Authors:** William MacAskill, Hannah Woodall, Claire Dorothea Nicholls, Kay Brumpton, Janani Pinidiyapathirage

**Affiliations:** 1 Griffith University Rural Clinical School, Toowoomba, Australia; 2 Rural Medical Education Australia, Toowoomba, Australia; 3 University of Southern Queensland, Toowoomba, Australia

## Abstract

**Introduction:**

Medical students learn to reflect to gain new insights into self and practice; however, allowing for reflection within a busy curriculum is challenging. In this study we embedded reflective writing prompts (RWP) into an existing assessment item, Online Submission of Case Reports (OSCAR), to investigate whether this minimalistic scaffolding intervention could develop students’ reflective capacity and increase their exposure to rural social determinants of health.

**Methods:**

This study is framed by ontological realism and informed by an interpretivist stance. Focus group transcripts (medical students and educators) were inductively analysed using thematic analysis. Written OSCAR reflections were analysed in a deductive top-down method to provide a contrasting perspective and triangulation.

**Results:**

Focus groups included 27 students, 10 educators, and 52 OSCAR reflections. Inductive analysis generated three themes: Scaffolded Learning, Affording Diverse Responses, and Maximising Learning Opportunities. Deductive analysis indicated that most students (87%) demonstrated lower-order thinking.

**Discussion:**

Most participants valued the impact of RWP on students’ learning. Though the RWP did not assist students to demonstrate higher-order thinking, they did increase the breadth of rural social determinants of health topics reflected upon by students, thereby increasing student knowledge of the impact of rural context on patient care.

## Introduction

**Figure 1:**
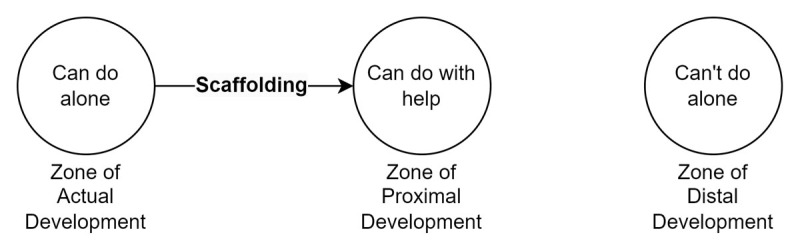
Scaffolding supports learners to develop new skills which are otherwise beyond their capabilities.

Reflective observation is a key component of experiential learning (learning by doing) and enables individuals to gain new insights into self and practice [1,2,3]. However, developing the skill of reflective observation requires support, particularly for students. Scaffolding refers to processes which enable novices ‘to solve a task or achieve a goal that would be beyond [their] unassisted efforts’ [4]. Scaffolds built into tasks assist learners to master skills in their zone of proximal development (Figure 1). Scaffolding can be incrementally reduced over time, allowing students to develop more advanced and independent skills [5]. In the clinical experiential learning context, scaffolding can assist medical students to reflect upon cognitive processes, thoughts, and feelings – ultimately improving their capacity to think critically and identify solutions to the complex problems which appear in clinical practice [6,7,8,9].

Scaffolded reflection also supports practitioners to make decisions based upon considered learnings from past experience [10,11]. Developing tools to enhance reflective capacity may therefore support students’ ability to critically assess patient presentations, understand patients’ perspectives and priorities, and provide holistic care.

Despite its importance, finding time to develop students’ reflective practice is challenging. Many medical curricula are overcrowded, which negatively effects students’ long-term learning and stress levels [12,13,14,15]. Embedding reflection in existing activities may therefore bring the benefits of enhancing reflective capacity while avoiding an increased curriculum burden. Additionally, embedding reflection in curriculum activities promotes reflection as part of day-to-day learning [16]. Published examples of embedded reflective activities include flash-card prompts [17], social media reflections [18], digital storytelling [19,20], and reflective debriefs [21,22].

In our context – a rural Australian Longitudinal Integrated Clerkship (LIC) – students complete an assessment task named the Online Submission of Case Reports (OSCAR) [23,24]. The OSCAR tasks are case-based learning activities during which students reflect upon one of their patient presentations, history, and examination, and then deliver their analysis and reflections as a report and presentation to their peers and educators. Within the OSCAR students are encouraged to reflect upon social determinants of health by exploring ethical, legal, professional, psychosocial, public health, cultural, and rural issues. Historically, many students in our program submitted no response or provided only cursory comments to this section. For metropolitan medical students to create effective treatment plans for rural patients they must first comprehend how rural social determinants of health impact the healthcare experiences of rural residents [25].

Recognising the value of diverse reflective approaches for medical students [26], the OSCAR task, with its multimodal approach and broad topics, was considered ideal for embedding reflective practice. Utilizing Dewey’s (1964) ideas on experiential learning [27,28], cognitive processes theorised by Bloom [29], and the concept of scaffolded instruction [30], we embedded scaffolds in the form of Reflective Writing Prompts (RWP) into the OSCAR task. We thereby aimed to support students to complete the reflective component of the OSCAR task and to improve students’ reflective capacity and understanding of social determinants of health in a rural context.

In this study we explore if RWP: 1, can be used to develop students’ reflective capacity; 2, can expand students’ knowledge of rural social determinants of health; and 3, are acceptable to students and educators. To answer these research questions this study utilized a combination of inductive and deductive analysis of focus group transcripts and completed student OSCAR reflections.

## Materials and Methods

### Context

This study was conducted with students and education staff in a rural clinical school that implements a LIC program in regional and rural Queensland, Australia.

### The OSCAR task

The OSCAR task encompasses a written comprehensive review of a patient’s history, presentation, and outcomes, and an exploration of social determinants of health. During their weekly education sessions at rural sites, students either presented OSCAR tasks or observed their peers’ presentations, followed by discussion with peers and educators. On average, students completed 12 OSCARs per year. To avoid curriculum overload, the OSCAR task was kept unchanged. However, students were provided with RWP to scaffold their reflections to two parts of the OSCAR task which explored social determinants of health from the perspectives of:

1. Ethical, legal, professional issues.
2. Psychosocial, public health, cultural and rural issues.

### Reflective Writing Prompts

Based upon insights from site educators and published studies such as Seymour and Watt [17] we developed reflective prompts to encourage students to explore the following topics: invisibility, autonomy, consent, multidisciplinary teams, confidentiality, mistakes, family, boundaries, personal value conflicts, saying no, uncertainty, rurality, distance, powerlessness, social determinants of health, barriers to care, communication, patient perspectives, vocabulary, strong emotions, culture, prescribing, procedures, and prognosis. A diverse array of prompts was provided to influence the depth and breadth of reflections generated [31] and to help students make connections between learning experiences, previous knowledge, unique rural healthcare dynamics, and different perspectives. Twenty-four prompt cards were provided, 11 relating to ethical, legal, professional issues (Appendix 1) and 13 relating to psychosocial, public health, cultural and rural issues (Appendix 2). Prompt cards contained one to four questions or instructions designed to support students to expand their knowledge of rural social determinants of health and to produce reflections using higher-order thinking. Students had discretion as to which prompts, and how many prompts, they used to aide their reflective writing.

### Methodology and research design

As the research focussed on students’ lived experiences, a realist approach to ontology informed by an interpretivist stance on knowledge and data was adopted. These perspectives underpinned a subjectivist research paradigm which posits that knowledge gains significance only through firsthand experience. These methodological choices were made to prioritise understanding participants’ firsthand experiences of the research and their reflected perspectives and practices of using the RWP.

Data collection methods employed in this study include the use of focus groups to collect students’ and educators’ perspectives on RWP in the OSCAR tasks and an analysis of a sample of written OSCAR reflections. Focus group transcripts were analysed inductively using thematic analysis while content analysis was applied with the aid of validated reflective assessment criteria to assess the written OSCAR reflections. Further detail on the study methods is provided in *Data collection and analysis*.

Griffith University Human Research Ethics Committee granted approval for this study (GU 2021/376).

### Participants

Participants included rural LIC students and their educators. The students were post-graduate medical students in their penultimate (third) or final (fourth) year of training and stationed in seven regional or rural towns categorised according to the Modified Monash Model (MMM) level as 2–5 [32]. These students had previously studied Reflective Learning (using the MaRIS model) as a graded unit of study within their medical course [33]. Educators were rural generalist doctors, specialist general practitioners or clinical nurse educators who served as student supervisors at the rural hospital sites. Including students and educators allowed exploration of RWP impacts from different perspectives including those of and about students, the student cohort, and curriculum implementation and delivery.

### Recruitment

Fifty-two medical students in the LIC program and their supervising educators were informed of the research study at routine education sessions. Invitations to participate were then sent by email and through an online curriculum application (Moodle). One reminder was sent via email. All research participants were provided with participant information sheets which fully disclosed the study’s objectives, methods, and approaches to maintaining confidentiality. Participants provided informed written consent prior to participating in research activities.

### Reflexivity

As some researchers were known to students and educators participating in the study, additional measures were taken to minimise perceived power imbalances between researchers and participants. WM, who was not involved in student grading, facilitated student focus groups and HW facilitated educator focus groups given her longer standing collegial relationship with these participants. Authors also considered the influence of their professional backgrounds, experiences, and prior assumptions during data extraction and analysis. WM has an education and physiology background; HW and JP are clinical researchers; KB is a general practitioner and experienced medical educator; CDN has an education background and specialist experience in qualitative research practices and pedagogical theories. WM and CDN approached data extraction and theme generation from a perspective free of expectations regarding what education in a clinical setting ‘should’ resemble. HW, JP, KB facilitated discussion of emerging codes and categories with the broader medical education team enabling new insights from those with experience in medical education delivery.

### Data collection and analysis

Data collection and analysis occurred in two parts and are described separately.

#### 1. Semi-structured focus groups

Within our methodological framework, semi-structured focus groups (Focus Group Guide: Appendix 3) served as the primary data collection method.

Five focus groups were run in total: one for Year 3 students, two for Year 4 students, one for medical educators, and one for nurse clinical educators. Focus groups were audio-recorded with a handheld device, transcribed using Sonix™ software (Sonix, United States), checked for accuracy and de-identified prior to analysis. Data was then imported into NVivo software (v1.7.2, QRS International, United States) to manage coding and analysis. Thematic analysis was conducted inductively using Braun and Clarke’s methods of thematic analysis and synthesis [34]. WM, HW, and CDN reviewed the full dataset. Initial codes and themes were developed by HW to ensure congruity and comprehensiveness. Codes and themes were further refined by HW, WM and CDN and approved by all authors.

#### 2. Written OSCARs

One randomly selected OSCAR from each of the fifty-two rural students was analysed.

Only OSCARs in which students had attempted reflection were included (e.g., blank responses and responses of ‘Not Applicable’ were excluded). The deductive coding was performed independently by HW and a research assistant, with discrepancies resolved through discussion. Using the Reflective Ability Scoring Rubric, a validated reflective assessment criteria [35], six levels of reflection were used (Table 1), from ‘Describes without reflecting’ (i.e., detailed description without reflection on action) through to ‘Integrates previous experience with current events and data to inform further action’ (i.e., analysing experience to specifically guide future action) [35]. Many OSCARS included more than one episode of reflection. The highest level of reflection within the OSCAR was recorded to indicate the maximum level of reflection attained by the student. The Reflective Ability Scoring Rubric does not define higher-order thinking. However, as the RWP used in the OSCARs were designed to stimulate higher-order thinking the six reflective levels of the Reflective Ability Scoring Rubric (Table 1) were aligned with the levels of Bloom’s Taxonomy [36]. Levels 1–3 aligned with lower-order thinking (e.g., knowledge retrieval), level 4 indicated students progressing towards higher-order thinking, while levels 5–6 would demonstrate that students were engaging higher-order thinking (e.g., thinking involving analysis and evaluation).

**Table 1:** Reflective Ability Scoring Rubric on Action Rubric. Table reproduced and amended from O’Sullivan et al. [34] under licensing conditions CC-BY-NC-SA. Cognitive verbs utilized in Bloom’s taxonomy are bolded to illustrate the convergence of the rubric with established educational theory [35,36].


BLOOM’S TAXONOMY	LEVEL	REFLECTION PERFORMANCE	SCORING GUIDELINES	ELABORATED GUIDELINES	EXAMPLES OF STUDENT OSCAR REFLECTIONS	HIGHEST LEVEL ATTAINED BY STUDENTS (%)

Demonstrates lower order thinking (recall, understand, describe)	1	**Describes** without reflecting	Narrative **description** of encounter but no evidence of reflection on action.	Very detailed story with some insight into behavior in the moment but **no further discussion** of behavior in retrospect.	Patient has decision-making capacity to provide consent.	3.7

	2	**Does not justify** lessons learned	**States** that lessons were learned but without explicit linkage to supporting evidence.	Vague reference to lessons learned without elaboration. **List** of lessons learned without linkage to evidence. **General** platitudes about optimal care **without** specific **linkage** to scenario.	I should have asked when the patient’s last cervical screening test was.	29.6

	3	Provides **limited justification** of lessons learned	Relies on personal assessment of lessons learned.	Personal **opinion** about lessons learned predominates. Little or no inclusion of external evidence as defined below.	Given the multiple co-morbidities this patient has if he were to contract influenza he would be at significantly higher risk of having a poor outcome hence the flu vaccine would be recommended – whilst he declined during his appointment it would be important to re-recommend at every appointment he attends	53.7

	4	**Includes evidence** of lessons learned	Includes external evidence of lessons learned.	External evidence **includes detailed feedback** from patients or professional associates, objective data on outcomes, and/or use of the literature	The mother requested antibiotics despite the GPs lack of clinical suspicion for such prescription. This prompted me to review the law surrounding whether a doctor has a duty to provide a particular type of treatment if not clinically indicated.	13.0

Demonstrates higher-order thinking (apply, analyse, evaluate, justify)	5	**Analyzes** factors from experience	Explicitly refers to prior experiences and describes how they inform own behavior in current situation.	**Reference to prior experience** can reinforce successful practices or **inform a change** in practice. Must meet criteria for level 4: even if **analyzes** factors from experience, cannot achieve this level without including external evidence of lessons learned.	N/A	0

	6	**Integrates** previous experience with current events and data to **inform further action**	**Analysis** including external evidence of lessons learned, relation to prior experience and **implications** for the future.	Must meet criteria for level 5 and also include a specific plan for the future including how success will be monitored.	N/A	0


## Results

### Overview

Twenty-seven medical students (Year 3 [Y3–S], *n* = 6, 25%; Year 4 [Y4–S], *n* = 21, 75%) and ten educators (medical educators [ME], *n* = 6, 86%; nurse educators [NE], *n* = 4, 80%) participated in the focus groups. No participants withdrew from the study. Three themes were generated from focus group data, *Scaffolded Learning, Affording Diverse Responses*, and *Maximising Learning Opportunities*.

One written OSCAR reflection from each student in the cohort (52 in total) was reviewed. Themes generated from students were similar regardless of year. Themes generated from educators were similar regardless of profession. Consequently, themes are drawn from, and are representative of, the full data set.

### Scaffolded learning

All participants identified that students found it difficult to commence the reflective portion of their OSCAR tasks. This difficulty was amplified when students were in their first clinical placement or when students perceived their OSCAR case as lacking interesting or exciting events. The use of RWP were seen as a beneficial scaffold for overcoming this writer’s block, particularly during the early stages of students’ first clinical placement.

‘I liked thinking [about the RWP] at the beginning of the year to kind of show me the range of things we could talk about and consider.’ Y3–S3

‘When they’re starting out with OSCARs, they tend to be descriptive because they don’t have the capacity [to reflect] … I wonder if that’s the most important time for them to engage in reflection?’ ME-2

Beyond facilitating the commencement of reflection, the RWP also acted as scaffolds for engagement in other types of learning. For instance, RWP provided a reflective lens which assisted students to engage in self-reflection, interrogate their thought processes, and develop their professional identity.

‘It’s my favourite bit of the whole OSCAR … I can actually learn about myself, learn about the patient, and learn about how to be a good doctor.’ Y3–S1

Additionally, the RWP supported students’ capacity to consider the perspectives of others. Students described increased understanding of how patients’ contexts and life experiences affect their healthcare journeys. Educators emphasized the positive impact of the RWP on students’ understanding of their colleagues’ and peers’ perspectives, motivations, and experiences.

‘I feel that being forced to reflect on that has translated into practice. And whenever I go into a patient’s room now, the first thing I think of is, what is going on in your life?’ Y3–S1

‘Everything else [in the OSCAR] is fact. This is just one student’s experience, experience or understanding, and it might not necessarily be the same as the person sitting beside them. I think that’s why it generates so much discussion. I definitely think it’s a good thing.’ NE-5

### Affording Diverse Responses

Students appreciated that the RWP afforded a broad set of topics for consideration and were particularly valuable when reflecting upon presentations which were ‘simple’.

‘I personally found [RWP] more helpful if you just didn’t know what to think about because you didn’t feel there were major issues.’ Y4–S4

The RWP were also useful where students lacked clinical experience, and thus deeper awareness, of the full complexities of their clinical cases.

‘Looking at a case [that is] clinically bare bones you might not ever think about if there was any confidentiality issues in that scenario … I think it gives them direction and helps them think about things.’ NE-2

This deepened awareness was perhaps best reflected by students who viewed the prompts as a resource they would access when they ‘didn’t know what to write’ [Y4–S4] or as a tool to support analysis in uncomplicated cases, ‘Ah, what do the prompts say?’ [Y3–S3].

‘I found them really useful because a lot of the time you’d have no idea what to write right now and it would help you. Sometimes it actually prompted me to something that did happen in the case that I could reflect on.’ Y4–S14

Some students felt the RWP did not assist them to write reflections on uncomplicated presentations, an opinion at times amplified by lower presentation rates in rural placement sites.

‘I’ve only seen one patient in the last three weeks that was critical care, so [the reflection] has to be on that.’ Y4–S5

‘It [RWP] doesn’t really solve the issue that if nothing happened in the case, there’s nothing really to reflect on and you’re kind of screwed.’ Y4–S3

Most students and educators noted that using RWP generated valuable discussions of a broader range of topics than was typical. This was seen as beneficial because it extended students’ focus from ‘Consent’ and the capacity of children under 16 years to provide consent according to the principle of ‘Gillick competence’ [37] [ME-5] to a more diverse range of factors influencing patient care and outcomes such as patients’ personal circumstances, students’ own biases, legal implications, and psychosocial factors.

‘It makes you go, “oh wow, look how many issues there actually are”.’ ME-5

‘My students made comments that when they got to the end of a case and they had no idea what to talk about, that they found these useful that they could go, “Oh, well, I’ll talk about this thing rather than talking about consent 5000 times”.’ ME-2

### Maximizing learning opportunities

The quality of the insights made by students through their written OSCAR reflections was explored using deductive coding (Table 1). These results indicate that most students were applying information (e.g., their experiences) towards learning for future scenarios, equivalent to level three of Bloom’s Taxonomy. A smaller proportion, approximately one-tenth, demonstrated reflections which approached higher-order thinking (Table 1, Level 4). These reflections indicated students’ ability to reflect analytically on their personal perception of the situation and to incorporate external information (e.g., feedback, guidelines, or literature) into the lessons learnt from their experiences. This result correlates with students demonstrating the fourth analytical level of Bloom’s Taxonomy where they display their ability to analyse information from multiple sources, evaluate and make future plans accordingly. Analysis of students’ reflections found no evidence of higher-order thinking as described in Table 1 or Bloom’s Taxonomy.

In focus groups participants provided suggestions on how the RWP could be more effectively utilized within the OSCARs to support reflection. One recommendation was to amalgamate the two reflective questions (i.e., 1, reflections on Ethical, legal, and professional issues; and 2, reflections on Psychosocial, public health, cultural, and rural issues) into a single reflective question. Students believed this change would allow for greater freedom in how they structured their reflections.

‘Having one [question] would significantly improve reflection, because most of the time there is … at least one thing to talk about. And the conjuring up of multiple things is the unpleasant part.’ Y4–S11

Some students further suggested that this amalgamated question should include scope for affective reflection (i.e., addressing social-emotional learning) and affective prompts in the RWP.

‘[RWP] could include some affective prompts as well.’ Y4–P8

These suggestions were mirrored by the educators who felt that the RWP should include ‘clinical and non-clinical prompts’ ME-2.

## Discussion

This study augmented OSCAR assessment tasks with RWP to provide additional opportunities for medical students to engage in reflective practice, develop higher-order reflective thinking, and gain knowledge of rural social determinants of health. Our findings indicate that the RWP are a useful learning tool for supporting engagement in reflective practice and that it is well received by students and educators. The RWP were found to be a useful writing scaffold, particularly in the early stages of clinical placement. RWP supported students’ ability to reflect upon and generate insight into a broader range of social determinants of health topics related to rural patient care. The RWP were not linked to increased workload and were viewed favourably by most participants. Embedding RWP into existing curriculum tasks may be a useful way of increasing opportunities for reflective practice and scaffolding quality reflection without contributing to curriculum overload.

The experience of RWP as a useful writing scaffold aligns with established views of educational theory, particularly around increasing rigour in student responses and scaffolding higher-order thinking [38]. For instance, Year 3 and Year 4 medical students are aware of social determinants of health but their ability to translate these concepts into a rural context is hampered by their limited practical experience of rurality. The RWP provide scaffolding to address barriers like these by narrowing the choices and presenting suggestions of how to respond; this reduces the complexity of the task and assists students to engage with a task in their proximal developmental zone [4,5]. Furthermore, the additional information provided on each prompt provides a clear example of what one could reflect upon, thus supporting students to develop independence from the RWP [5,30]. This theoretical viewpoint is supported by comments of educators who noted that the RWP were more useful earlier, rather than later, in students’ clinical placements.

The RWP were successful in expanding the breadth of topics reflected upon by students in their OSCAR reflections. This indicates that the RWP were a useful tool for broadening students’ understanding of patient priorities, holistic care, and the impact of the doctor on care provided to patients in rural communities. It was also hoped that the RWP would develop the reflective capacity of students. However, we did not demonstrate that the RWP assisted students to use higher-order thinking in their reflections. Therefore, in their present iteration, our RWP may be best suited for increasing the breadth, rather than depth, of reflections completed by medical students. Contemporary research indicates that engagement and the depth of reflection achieved in writing tasks is substantially influenced by the construction of the writing prompts themselves [31] and the provision of clear instructions [39]. In particular, student reflections that demonstrated greater higher-order thinking (analysis, evaluation and justification) tended to originate from prompts which: 1, included clear learning goals; 2, specified the type of writing to be produced (e.g., revise, plan, process, draft, essay, reflect); 3, used words which encouraged students to reflect on themselves (e.g., I) and on others (e.g., student, peer, audience, context), and 4, asked students to reflect on something specific [31]. A comparison of our RWP to the above recommendations indicates there is room for improvement in their design. For instance, RWP could include a greater emphasis on reflecting on self and others (i.e., beyond the patient) and could incorporate more non-clinical learning outcomes as learning goals (e.g., what have you learned from this experience and how does it relate to your understanding of multidisciplinary team theory?). Such changes may assist students to not only reflect upon broader topics relating to medicine and patient care, but also develop more rigorous reflective writing skills and reflective capacity.

Limitations of the study include the relatively lower recruitment of Year 3 students. Our findings suggest that RWP are most useful when students were new to clinical practice, however this observation was reported primarily by Year 4 students who had progressed beyond the initial stages of their clinical placements. A further limitation of this study was an absence of data on the contributions of the RWP to the oral presentations of OSCARs and to subsequent discussions of the case, as these verbal exchanges are further opportunities for students to demonstrate their reflective thinking.

This study utilised a minimalistic intervention which provided no additional instruction on reflection to students (beyond the RWP themselves), instead relying on students having learned the fundamentals of reflection in other units of study. Given participants demonstrated no higher-order thinking in their OSCAR reflections, additional support or instruction may be required alongside the RWP to effectively support students to further develop their reflective capacity. Though further investigation is needed to identify if embedding RWP with additional support or instruction can assist medical students to reflect more deeply, this study showed embedded RWP can expand the breadth of rural social determinants of health topics reflected upon by students.

The RWP were positively received by most participants and no negative impacts from their implementation were raised – indicating that embedding RWP in existing tasks may be a simple way of incorporating additional reflective practice into medical curricula. Most participants were supportive of ongoing implementation and refinement of the RWP, viewing them as useful learning tools, and indicated that the RWP made it easier to engage in reflection. RWP may be a valuable addition to medical education assessments, and if suitably designed, could support students to develop their reflective skills while engendering a broader appreciation of the many factors which influence patient care and outcomes. To summarise the RWP in the words of one student ‘they don’t take any value from the assessment – they only add to it’.

## Data Accessibility Statement

The data that support the findings of this study are available from the corresponding author, William MacAskill, upon reasonable request.

## Appendices

**Appendix 1:** Ethical, Legal, Professionalism Reflective Writing Prompts.

**Invisible:** How can patients be rendered invisible in the health system? E.g. a patient referred to by condition or room number rather than name, holding a conversation over a patient without including them.

**Family:** Consider the importance of family to patient’s health and wellbeing. How was this facilitated or obstructed during their interaction with the health system?

**Multidisciplinary teams:** How many professions were involved in the care of the patient in this interaction? Pick one and reflect on how their input contributes to the patient’s recovery/wellbeing.

**Confidentiality:** Where has confidentiality been limited or breached in this case? Remember to consider more ‘subtle’ examples – a hallway discussion/consult or a sensitive discussion held in a four bed bay.

**Consent:** Reflect on a situation where consent has been sought (e.g. for surgery, a procedure, an examination, or for student involvement). How informed was the consent? What can you learn from this – positively (what to replicate) and negatively (what to avoid)?

**Autonomy:** In this case, reflect on a situation where the patient has declined or stopped treatment. Did the team agree with the decision and how did they navigate this? Consider the patient perspective – what reasons did they have for their decision?

**Mistakes:** Reflect on where a mistake has been made in this case. How was it dealt with? What was the impact on the patient/on the team/on the student?

**Boundaries:** Reflect on the professional boundaries placed by yourself or a colleague/supervisor in this case. What challenges arise in balancing emotional engagement with patients with professional distance?

**Personal value conflicts:** Reflect on how a practitioner’s personal values could interfere with a patient’s care. E.g. what if a patient’s illness is perceived to be their “own fault” due to smoking, weight, drink-driving. How can this impact care, how can practitioners manage this?

**Saying No:** Reflect on where you or another member of the team have had to say no to a patient (e.g. request for medication, no longer able to drive etc.). What challenges did this raise and how were they managed?

**Uncertainty:** Reflect on the issue of uncertainty in this case. This may be team uncertainty (e.g. diagnosis unclear) or student uncertainty (e.g. how to respond when you can’t answer a patient’s question). How was this managed, how was it expressed to the patient?

**Appendix 2:** Psychosocial, Public Health, Cultural, and Rural Issues Reflective Writing Prompts.

**Rurality:** How may this case have been different if it occurred in an urban rather than a rural location? Consider potential positive and negative differences.

**Distance:** How did distance impact healthcare for this patient/in this case?

**Powerless:** Identify factors which make this patient feel powerless (these may be social, cultural, economic, education). Consider how this impacts on their health.

**Social determinants:** Identify factors influencing the patient’s health that are out of their control. Consider the role of the doctor/student in advocating for the patient when it comes to these factors.

**Barriers to care:** Reflect on barriers to accessing care that exist for this patient. How these could be reduced?

**Good/bad communication:** Reflect on an example of communication from this case that stands out as particularly good or bad? What made it so good or bad? What can you learn from this?

**Vocabulary:** Consider the vocabulary used with this patient. Was it appropriate/understood by the patient? Were loaded terms (e.g. non-compliant) or jargon used? How may this have affected the interaction?

**Patient’s perspective:** Reflect on the patient’s perspective or theories about their illness. What worries them most? Did this align with what worried you most? What challenges did this cause?

**Strong emotions:** How were negative emotions (e.g. anger, grief, frustration) from a patient or family member dealt with in this case? How did the team manage the situation? What were the reasons/patient’s perspective behind the emotions?

**Culture:** How did culture impact on this patient’s health, wellbeing or care? Consider the culture of the patient, the doctor and the student.

**Rational prescribing:** Reflect on the NNT for one medication prescribed for this patient. What does this mean for the patient? Was this discussed with them?

**Procedures:** Reflect on a procedure (diagnostic/screening) being offered to this patient. What are the risks? How likely are false negatives/positives from this test? How does this impact your advice to the patient and interpretation of this test?

**Prognosis –** Research the prognosis/possible outcomes for the patient – may be relevant in the case of a life-limiting illness or in other conditions (e.g. likelihood of perforated TM healing or dislocation recurring). Was this discussed? How does this affect the advice you may give the patient?

## Appendix 3: Focus Group Guides

### Focus Group Outline (Educators)

This is an outline of the types of topics to be covered in the focus group, with prompts for discussion where needed. The intention is to encourage discussion and gauge educator responses to the reflective learning portion, with this document used as a guide only.

Focus Group Questions:

1. What is your view on reflection or reflective learning activities for students?
  - Is it important for students? Why/why not?
  - What are the benefits/disadvantages/challenges?
  - What do you see as its purpose in medical education?
2. The final two questions of the OSCARs require students to engage in reflective learning. Can you tell me about your experience with the reflective questions in the OSCARs presented by Griffith University Medical Students?
  - What is the quality of the reflections?
  - Do students find these difficult? If so, what are the challenges?
  - To what extent is this a focus of the OSCAR presentations?
3. This year, students were provided with reflective prompts to assist and guide their reflections.
  - Did students use these? Did you receive any feedback?
  - Was there any impact on the student reflections? If so – what impact?
  - Should they continue to be used and if so, can you recommend any changes?
4. How do you think we can best help students to learn reflective practice skills?

Close of session:

Thank you for participating in this focus group. If you have any questions or concerns related to this project at any time, please contact the research team or the Griffith University Ethics Office. The contact details for these groups are provided in your information sheet.

Following completion of this project, a summary of the study findings will be developed. If you would like to receive a copy of this summary, please provide your email address to the group facilitator. This email address will be stored securely and separate to the study data, and used only to circulate the summary at the conclusion of the project.

### Focus Group Outline (Students)

This is an outline of the types of topics to be covered in the focus group. The intention is to encourage discussion and gauge student responses to the reflective learning portion, with this document used as a guide only.

Focus Group Questions:

1. What do you think of when you think of reflection or reflective learning activities?
  - What benefits can you see?
  - What disadvantages can you see?
2. What is the purpose of reflection for a medical student?
3. Tell us about your experience of these types of activities in the past
  - What helps/makes it more useful?
  - What make it less useful?
4. This year you had to answer some reflective questions as part of your OSCARs (the last two questions).
  - Tell me about your experience with these questions
  - If prompts needed
    - Was it difficult to think of topics to reflect on?
    - Were they difficult to answer?
5. You were also given a list of prompts to use to help you answer these questions
  - Did you use these?
  - What was your experience of these?
    - Were they helpful – if so, how?
    - If not – why?
    - How can we make them better?
6. How do you think we can best help students to learn reflective practice skills?

Close of session:

Thank you for participating in this focus group. If you have any questions or concerns related to this project at any time, please contact the research team or the Griffith University Ethics Office. The contact details for these groups are provided in your information sheet.

Following completion of this project, a summary of the study findings will be developed. If you would like to receive a copy of this summary, please provide your email address to the group facilitator. This email address will be stored securely and separate to the study data, and used only to circulate the summary at the conclusion of the project.
